# Predictions of cervical cancer identification by photonic method combined with machine learning

**DOI:** 10.1038/s41598-022-07723-1

**Published:** 2022-03-08

**Authors:** Michał Kruczkowski, Anna Drabik-Kruczkowska, Anna Marciniak, Martyna Tarczewska, Monika Kosowska, Małgorzata Szczerska

**Affiliations:** 1 Faculty of Telecommunications, Computer Science and Electrical Engineering, Bydgoszcz University of Science and Technology, Al. prof. S. Kaliskiego 7, 85-796 Bydgoszcz, Poland; 2 Department of Obstetrics, Gynaecology and Oncology, Faculty of Medicine, Ludwik Rydygier Collegium Medicum in Bydgoszcz, Nicolaus Copernicus University in Toruń, 85-094 Bydgoszcz, Poland; 3 Department of Forensic Medicine, Department of Molecular and Forensic Genetics, Faculty of Medicine, Ludwik Rydygier Collegium Medicum in Bydgoszcz, Nicolaus Copernicus University in Toruń, 85-094 Bydgoszcz, Poland; 4 Department of Metrology and Optoelectronics, Faculty of Electronics, Telecommunications and Informatics, Gdańsk University of Technology, 11/12 Narutowicza Street, 80-233 Gdańsk, Poland

**Keywords:** Optical sensors, Data processing, Machine learning, Cancer, Biomedical engineering

## Abstract

Cervical cancer is one of the most commonly appearing cancers, which early diagnosis is of greatest importance. Unfortunately, many diagnoses are based on subjective opinions of doctors—to date, there is no general measurement method with a calibrated standard. The problem can be solved with the measurement system being a fusion of an optoelectronic sensor and machine learning algorithm to provide reliable assistance for doctors in the early diagnosis stage of cervical cancer. We demonstrate the preliminary research on cervical cancer assessment utilizing an optical sensor and a prediction algorithm. Since each matter is characterized by refractive index, measuring its value and detecting changes give information about the state of the tissue. The optical measurements provided datasets for training and validating the analyzing software. We present data preprocessing, machine learning results utilizing four algorithms (Random Forest, eXtreme Gradient Boosting, Naïve Bayes, Convolutional Neural Networks) and assessment of their performance for classification of tissue as healthy or sick. Our solution allows for rapid sample measurement and automatic classification of the results constituting a potential support tool for doctors.

## Introduction

Cervical cancer is one of the most common cancers worldwide^1,2^. Every year around the world, cervical cancer is diagnosed in about half a million women, including about 2.5 thousand in Poland^3^. The incidence and mortality of cervical cancer have been dramatically reduced by screening programs^4^. However, in many cases diagnoses are still dependent on the doctor’s subjective interpretation, creating a strong need for solutions supporting them^5^.

Particularly important in the diagnosis of cervical cancer is the precise determination of the depth of neoplastic lesions, which is of clinical importance during cervical cancer management procedures) in order to correctly define the margin of pathological changes. Commonly used measurement methods such as colposcopy, visual inspection with acetic acid and Lugol's iodine are limited by the subjective judgment of the examiner and the lack of reliable measurement calibration standards^6^. The imprecise definition of the type of neoplastic lesion may lead to far-reaching consequences such as extended diagnosis time, high treatment costs, patient's exposure to unnecessary procedures, and in extreme cases even the patient's death. The main cause of the premalignant changes in the cervical epithelium is associated with the infection of HPV—Human Papilloma Virus^7,8^. Although there are approximately 100 types of HPV virus, only several create a high risk.

Since cervical cancer proper diagnosis is of greatest importance, several approaches aiming at its improvement were proposed and are still being developed. Most popular technique involves biopsy, imaging^9^ and doctor’s evaluation. The imaging gives many opportunities for data processing and analysis which results can support doctors during the diagnosis stage. A deep learning-based system for detection and classification of cancerous cells based on convolutional neural networks (CNN) was presented^10^. With the extreme learning machine-based classifier accuracy of 99.7% (detection) and 97.2% (classification) were achieved for input images. A dedicated pipeline was developed to automatically detect and classify cervical cancer from cervigram images^11^. The solution involves two pre-trained deep learning models and CNNs, assuring fast and accurate results. Automatic segmentation and classification by fuzzy C-means (FCM) clustering technique showed accuracy of 93.78% and 99.27% for 7-class and 2-class problems^12^. The deep learning method using stacked autoencoder—softmax model allows for dataset dimension reduction and reaching classification accuracy of 97.25%^13^.

An approach using Support Vector Machine (SVM) allows achieving an average accuracy of up to 90%, sensitivity of nearly 100% and specificity of 83%^14^. Moreover, the computation performance can be improved by reducing the number of factors to 8 variables in case of SVM-RFE (recursive feature elimination) and SVM-PCA (principal component analysis). However, SVM does not perform well in case of large datasets and the training is relatively slow.

Most of the presented techniques show satisfying performance in accomplishing their tasks and the majority of algorithms are providing great accuracy^15^. However, commonly used CNNs require a big database for the training of the models which may be a challenge in case of medical data. It is also worse in terms of time performance in comparison to the classical algorithms. Such algorithms assure high scores of classification accuracy, i.e. Random Tree, Random Forest, Instance-Based K-nearest neighbor giving over 98%^16^.

As the major approach involves image processing, we propose a simpler solution in terms of data acquisition, processing and overall data size reduction. In this paper, we propose the fusion of the most dynamically developing technologies: optical sensing and machine learning techniques^17–19^. With a fast, reliable and non-destructive optical method, we can investigate the biological sample and then analyze the acquired data with dedicated software^20,21^, allowing for auto-identification of neoplastic cervical lesions which will be invaluable support for doctors at the stage of initial diagnosis^22,23^. The identification will be based on refractive index values of measured tissues.

The refractive index is one of the most important physical properties characterizing materials. In case of biological tissues, it is highly correlated with the morphological features including the cell density and the nuclear-cytoplasm ratio. Based on cervical cancer’s state of the art^24^, refractive index of normal cells and cancerous cells are different, hence refractive index changes constitute a basis for relatively easy differentiation between the normal and cancerous cells^25^. Table 1 presents typical refractive index values obtained for both healthy and sick cervical cells.

**Table 1:** Associated refractive index values of cervical cells at different neoplastic progression.

Cell type	Basal	Midzone	Superficial
Normal	1.387	1.372	1.414
Cancer	1.426	1.404	1.431

In this study, we propose a method of preliminary cervical cancer identification based on a prediction algorithm, taught on data obtained from low-coherence measurements of certified refractive index liquids. We have measured and analyzed samples within the range of actual refractive index values for healthy cervical tissues and neoplastic lesions. The acquisition and preparation of datasets, machine learning process and results of the investigation are described. To date, no research applying machine learning for peculiar analysis of low-coherence data obtained for various refractive indices was reported. Our approach allows for fast and reliable analysis of such data and their classification, which is the starting point for the development of a system able of the initial identification of neoplastic cervical lesions. This can be a helpful tool for doctors greatly impacting and improving the effectiveness of early cervical cancer diagnosis.

## Methodology

The classification of cervical intraepithelial neoplasia (CIN) is based on a histological evaluation that differentiates three advancement stages: CIN1, CIN2, CIN3^26^. The grade of dysplasia is the proportion of cervical changes in the epithelium. CIN1 has a low potential for progression to malignancy. CIN1 is confined to the basal one-third of the epithelium. CIN2 has more marked nuclear abnormalities than CIN1. The dysplastic cellular is observed to the lower of two-thirds of the epithelium. The CIN3 occurs if the atypical cells are found in all layers of the epithelium. The characteristic features are a low potential for malignancy and a high potential for regression. The L-SIL (Low-grade Squamous Intraepithelial Lesion) corresponds histologically to CIN1. The H-SIL (High-Grade Squamous Intraepithelial Lesion, CIN2 and CIN3) has a higher potential for progression and lower potential for regression.

**Figure 1:**
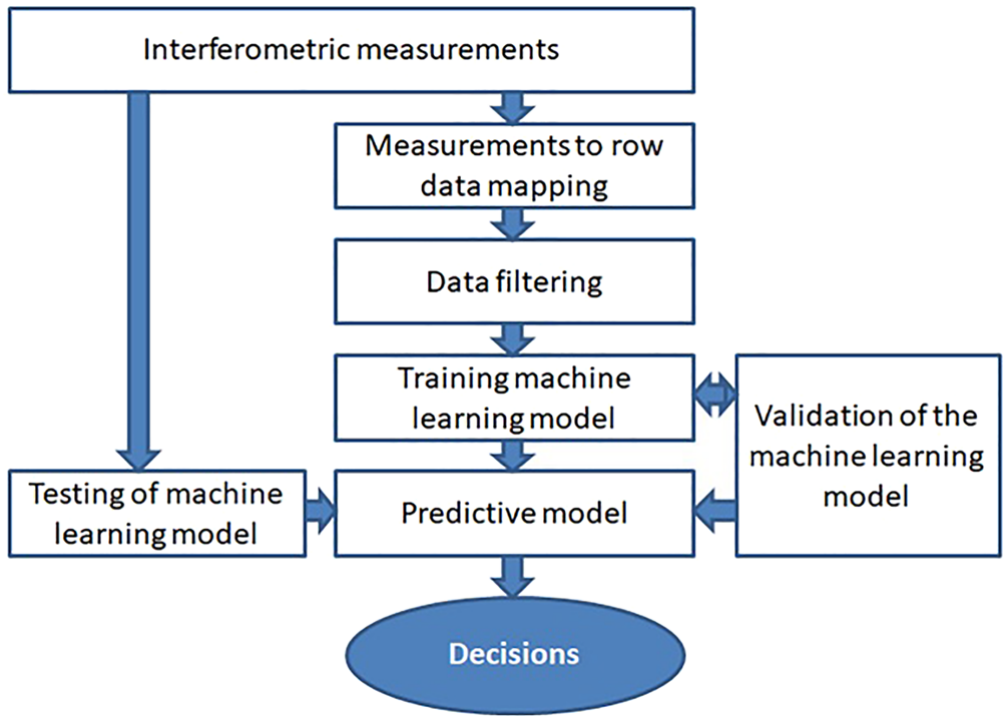
Methodology workflow.

The main goal of the cervical cancer identification method is to detect neoplastic lesions according to the designed methodology as shown in Fig. 1.

The proposed methodology includes four relevant modules: low coherence interferometric measurements, data preprocessing (row mapping, filtering), training of supervised machine learning model and testing the built predictive model.

Based on a literature study, the assignment of individual samples with known refractive index values to two classes (healthy or cancer) was defined^27,28^. The predictive capabilities of selected supervised machine learning algorithms were built and analyzed to select the optimal classification model. Moreover, the proposed method was tested on the basis of completely new test datasets that were not involved in the training process. It should be noted that the cancer is diagnosed when the basal membrane is invaded due to differences in treatment. However, the evaluation of the refractive index should be correlated with the identification of the basal layer. Therefore an essential element of the elaborated method is sensitivity to the Fabry–Perot interferometer length changes. This parameter corresponds to the depth of the cervical epithelium of the measurement sample that determines the grade of dysplasia.

### Dataset acquisition

The optical determination of refractive indices of the investigated liquids was performed in a Fabry–Perot interferometer. The measurement setup was built in a reflective configuration using fiber-optic technology. The components of the system were a superluminescence diode (SLD-1550-13-, FiberLabs Inc., Fujimino, Japan), an optical spectrum analyzer (Ando AQ6319, Yokohama, Japan), a 2 × 1 optical coupler (Lightel, Renton, Washington, USA) and a micromechanical stand. The light source operated at the central wavelength of 1550 ± 20 nm with a spectral width of 35 nm. The Fabry–Perot resonance cavity was formed by the polished fiber end-face and a silver mirror^29,30^.

The light from the light source was guided through the fiber to the cavity. Partial reflections occurred at the two boundaries: fiber end-face/medium and medium/silver mirror. The reflected light beams interfered giving a signal recorded by the optical spectrum analyzer. The phase shift between interfering beams is dependent on their optical path difference (which is influenced by the geometrical path length and refractive index of the medium) according to the following formula^31^:

1 $$\mathrm{\varphi }=\frac{4\mathrm{\pi nl}}{\uplambda }$$

where ϕ—phase shift, n—refractive index, l—geometrical path length, λ—wavelength.

In our investigation, the geometrical path length difference (the width of the resonance cavity) was constant throughout the whole measurement process, hence the refractive index change was the only variable impacting the acquired signal^32^.

For precise measurements of the refractive index of liquids, we used the Certified Refractive Index Liquids by Cargille^®^ (Cargille Labs, Cedar Grove, USA). The investigated liquids were characterized by refractive indices in the range of 1.3–1.5 with a step of 0.01. The choice of this measurement range was based on the values of refractive indices of healthy and diseased tissues^33–35^. The range was extended to include inter-individual differences and assure a larger dataset for algorithm learning. This way, the results obtained by the proposed method can be directly translated into biological tissues. In this article, we refer to each oil using the label value (measured for 589.3 nm in 25°C) for clarity. However, the data analysis takes into account the nominal values given in datasheets for the wavelength equal to 1550 nm, as the source used in experiment^36^.

The highest signal contrast of V = 0.9956 was obtained for the cavity length equal to 280 µm. The reference signal was acquired to control the intact cavity setting. Next, 30 µL of the liquid sample with a known refractive index was introduced into the cavity. The optical spectra were recorded and the cavity was cleaned. The whole procedure was then repeated for all liquids (a total of 10 spectra for each sample).

### Dataset preparation

**Figure 2:**
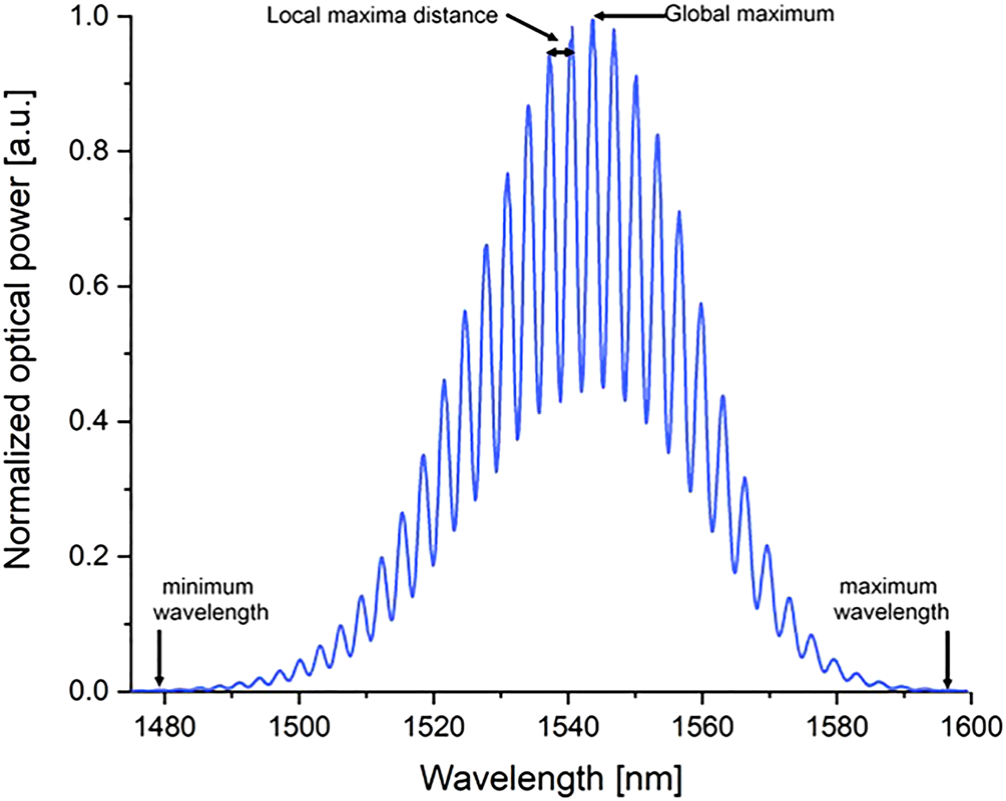
Sample interferogram.

Interferograms obtained in accordance with the adopted methodology were the basis for further analyzes. 210 interferograms were taken for analysis, each data consists of two columns representing the wavelength and the optical power of the signal. The representative signal is shown in Fig. 2. Furthermore, a theoretical interferogram for comparative analyzes was generated based on the following formula^37^:

2 $$T=1+\mathrm{cos}(\frac{4\pi n l}{\lambda })$$

where: n—refractive index, l—cavity length, λ—wavelength.

The main step of the preprocessing was mapping the measurement data to the feature vector—this way we obtained a dataset adapted to the training supervised learning model. The mapping process was based on 18 procedures in order to generate an 18-feature row dataset for each interferometric signal. In other words, the data enrichment techniques described in Table 2 were used. The interferogram was filtered with a threshold that represents a percentage of the global maximum. A part of signal rejected from the analyses—by multiplication of global maximum and threshold noises were eliminated. A noisy part of the signal was eliminated by the multiplication of a global maximum and a threshold value.

**Table 2:** Selected features description.

Symbol	Feature	Description
F1	Number of local maxima	Extraction of a list of local maxima in considered interferogram
F2	Global maxima	Maximum value from the local maxima list
F3	Threshold	A variable used to filter amplitude to smooth the signal (e.g. 5% of global maxima)
F4	Amplitude normalization factor	Used to rescale experimental plot compared to the simulation one, due to different ranges of amplitude; factor was calculated as shown in Eq. 2
F5	Local maxima distance–average	Average wavelength distance between the local maxima
F6	Local maxima distance–maximum	Maximum wavelength distance between the local maxima
F7	Local maxima distance–minimum	Minimum wavelength distance between the local maxima
F8	Local maxima distance–median	Median wavelength distance between the local maxima
F9	Dissimilarity measure	Dissimilarity between simulated and experimental interferogram (integral calculated with the use of Simpson rule as shown in Eq. 3)
F10	Chart axial shift	Global maxima shift between simulated and experimental interferogram
F11	Roots mean squared error (RMSE)	Difference between the simulation plot and the experimental data
F12	Cavity length	Value read from the configuration of the measuring set (Fabry–Perot cavity)
F13	Minimum wavelength	Minimum value for wavelength parameter
F14	Maximum wavelength	Maximum value for wavelength parameter
F15	Amplitude	Difference between maximum and minimum y value, where y is representative of amplitude column from input data
F16	λ0	Wavelength for maximum amplitude
F17	λ0 for theoretical signal	Wavelength for maximum amplitude (for base signal)
F18	Target variable	1—cancer, 0—healthy

**Figure 3:**
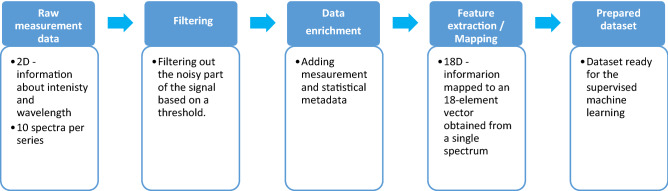
Flowchart of data preprocessing.

Each row in the dataset represents one sample and consists of 18 columns. Each column is representative of one from selected features. The target variable was assigned based on refractive index value: refractive indices between 1.30 and 1.38 were assigned as ‘healthy’ tissue while refractive indices between 1.39 and 1.50 were labeled as ‘sick’ tissue. The dataset was balanced, consisting of 43% of healthy samples and 57% of sick representatives. The flowchart of data preprocessing is presented in Fig. 3. Prepared dataset allowed to build a machine learning model based on selected supervised learning algorithm.

The following formulas were introduced into preprocessing procedure in order to estimate the distortion of the measurement interferogram with relation to the theoretical interferogram. Factor f is responsible for the fit of the theoretical signal amplitude to the measured interferogram as shown in Eq. 3.

3 $$\mathrm{f}=\frac{\mathrm{ssmax}}{\mathrm{global \,max}}$$

where: global max—global maximum for measured signal, ssmax—maximum value for simulation signal. Finally, the distortion level D of the measurement interferogram is calculated numerically using the surface area below the interferometric signal as shown in Eq. 4.

4 $$\mathrm{D}=\left|\frac{1-(\mathrm{area}\_\mathrm{exp }\times \mathrm{ f})}{\mathrm{area}\_\mathrm{sym}}\right|$$

where: area_sym—integral under the curve of the simulation plot, area_exp—integral under the curve of the plot of experimental data, f—factor.

**Figure 4:**
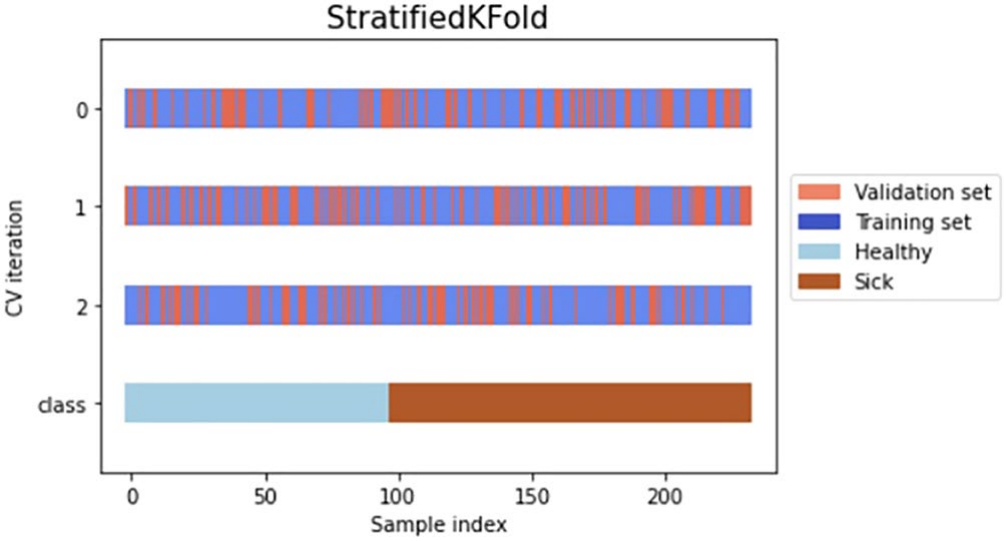
Graphical representation of Stratified 3-Fold Cross Validation on a prepared dataset.

Before the model training process began, the k stratified fold cross-validation method was used to divide the data into the validation and training dataset. We have selected k equals 3 in order to avoid the negative influence of overfitting phenomena with reference to the dataset size. Too large k-value means that only a low number of sample combinations is possible, thus limiting the number of iterations that are different. It should be noted that stratified sampling is a sampling technique where the samples are selected in the same proportion (by dividing the population into groups called ‘strata’ based on characteristics) as they appear in the population as shown in Fig. 4. The value of k was chosen experimentally from odd numbers set in the range from 3 to 9, due to the fact that each of the considered values of k, quite similar cross-validation results were obtained. On the other hand, the smaller the k value, the shorter time of obtaining cross-validation results.

Cross-validation is a resampling procedure, which is used to evaluate machine learning models on a limited data sample. Its main goal is to randomly divide data into a given number of sets on which the machine learning model is later tested. The obtained dataset statistics are presented in Table 3.

**Table 3:** Dataset statistics (coef—the coefficient of the independent variables and the constant term in the equation).

Symbol	coef	std error	test statistic t	P >|t|	[0.025	0.975]
F1	− 5.8e + 04	2.22e + 05	− 0.262	0.794	− 4.96e + 05	3.8e + 05
F2	− 0.0012	0.001	− 2.161	0.032	− 0.002	− 9.98e− 05
F3	− 0.0159	0.035	− 0.448	0.654	− 0.086	0.054
F4	− 0.0646	0.033	− 1.965	0.051	− 0.129	0.000
F5	6.203e + 04	2.33e + 05	0.266	0.791	− 3.99e + 05	5.23e + 05
F6	− 0.1583	0.654	− 0.242	0.809	− 1.450	1.133
F7	− 0.0221	0.064	− 0.345	0.731	− 0.148	0.104
F8	− 0.0091	0.035	− 0.264	0.792	− 0.077	0.059
F9	0.5103	1.621	0.315	0.753	− 2.689	3.710
F10	− 58.0557	362.914	− 0.160	0.873	− 774.423	658.312
F11	0.0962	0.051	1.872	0.063	− 0.005	0.198
F12	0.0167	0.093	0.180	0.858	− 0.167	0.200
F13	− 0.0057	0.022	− 0.264	0.792	− 0.049	0.037
F14	2.6056	0.805	3.238	0.001	1.017	4.194
F15	0.0323	0.094	0.346	0.730	− 0.152	0.217
F16	0.0004	0.000	0.775	0.439	− 0.001	0.001
F17	7.937e− 07	3.01e− 06	0.264	0.792	− 5.15e− 06	6.73e− 06
F18	− 5.8e + 04	2.22e + 05	− 0.262	0.794	− 4.96e + 05	3.8e + 05

### Machine learning

Referring to reported research where similar analytical problems were solved^38–43^, four algorithms were selected for further analysis: Random Forest (RF), eXtreme Gradient Boosting (XGBoost), Naïve Bayes (NB) and Convolutional Neural Networks (CNN). It should be noted that the use of well-known algorithms in the combination with the novel methodology of data preprocessing^44^ and enrichment is an unprecedented approach in the analysis and prediction of optical properties of measured substances. For each algorithm, optimal parameters were selected experimentally.

Random Forest^45,46^ and eXtreme Gradient Boosting^47,48^ classifiers utilize ensembles of classifications are receiving increased interest. Ensemble learning algorithms use the same base classifier to produce repeated multiple classifications of the same data or use a combination of different base classifiers to generate multiple classifications of the same data or to target different subsets of the data^49^. The collection of multiple classifiers of the same data are combined using a rule-based approach (such as maximum voting, product, sum or Bayesian rule) or based on an iterative error minimization technique by reducing the weights for the correctly classified samples (e.g. boosting). Ensemble learning techniques have higher accuracy than other machine learning algorithms because the group of classifiers performs more accurately than any single classifier, and utilizes the strengths of the individual group of classifiers while the classifier weaknesses are circumvented. Whereas Naïve Bayes classifier is a simple probabilistic classifier based on applying Bayes' theorem with strong (naïve) independence assumptions between the features^50^. They are among the simplest Bayesian network models but coupled with kernel density estimation, they can achieve higher accuracy levels^51^.

CNN is a biologically inspired deep learning algorithm, which consists of multiple layers including convolutional layer, non-linearity layer, pooling layer and fully-connected layer^52^. The processing units are arranged to model high level abstraction of data^53^. CNNs use relatively little pre-processing in comparison to other image classification algorithms, however, their main drawback is tendency to data overfitting. Neural Networks are widely used in data analysis, including processing of medical data^54^.

The first algorithm we tested was RF, where the following parameters were selected: n_estimators—100, criterion—gini, min_samples_split—2, min_samples_leaf—1. To test the possibility of improving the RF results, an XGBoost algorithm was used and the following parameters were selected: booster—gbtree, learning_rate—0.3, min_split_loss—0, max_depth—6 and sampling_method—uniform. As a part of the application of a different approach to classification, an NB algorithm was used. Following parameters were selected: priors—None, var_smoothing—1e−9. Finally, we used algorithm well-known in bioengineering—Convolutional Neural Networks (CNN). Following parameters were selected: 3 layers (32 units, 16 units and 1 unit), activation functions (rectified linear and sigmoid) and number of epochs—200.

## Results

Since the presented problem can be treated as binary classification, confusion matrices^55,56^ were used to evaluate and compare the ML-based methods. Four measures were defined as follows:

TP—true positives—cancer tissue classified as cancer;

FP—false positives—healthy tissue classified as cancer;

FN—false negatives—cancer tissue classified as healthy;

TN—true negatives—healthy tissue classified as healthy.

**Figure 5:**
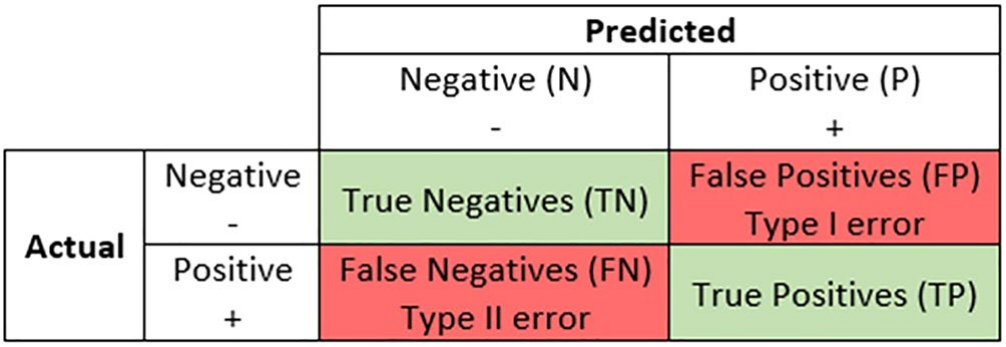
A graphical representation of evaluation measures: True Positives, False Positives, False Negatives, True Negatives.

A graphical representation of these measures is presented in Fig. 5.

In order to reliably evaluate the predictive ability of the model, we introduce the following classifier evaluation metrics: Accuracy (Eq. 5), Precision (Eq. 6), Recall (Eq. 7) and F1-score (Eq. 8).

5 $$\mathrm{Accuracy}=\frac{\mathrm{TP}+\mathrm{TN}}{\mathrm{TP}+\mathrm{FP}+\mathrm{TN}+\mathrm{FN}}$$

6 $$\mathrm{Precision}=\frac{\mathrm{TP}}{\mathrm{TP}+\mathrm{FP}}$$

7 $$\mathrm{Recall}=\frac{\mathrm{TP}}{\mathrm{TP}+\mathrm{FN}}$$

8 $$\mathrm{F}1=\frac{2(\mathrm{Precision} \cdot \mathrm{Recall})}{\mathrm{Precision}+\mathrm{Recall}}$$

The use of these metrics provides us information not only about the accuracy of the classification but especially important properties like sensitivity and specificity and the model's insensitivity to overfitting and underfitting. All measures used in those equations were mentioned above (TP, FP, FN and TN). The obtained results are presented in Table 4.

**Table 4:** Classification results.

Classifier	Fold	Accuracy	Precision	Recall	F1-score
Random Forest	1	0.97	0.97	0.98	0.97
	2	1.00	1.00	1.00	1.00
	3	1.00	1.00	1.00	1.00
Validation:		0.91	0.91	0.92	0.92
XGBoost	1	1.00	1.00	1.00	1.00
	2	1.00	1.00	1.00	1.00
	3	1.00	1.00	1.00	1.00
Validation:		0.89	0.90	0.90	0.89
Naïve Bayes	1	0.96	0.96	0.96	0.96
	2	0.95	0.95	0.95	0.95
	3	0.97	0.97	0.97	0.97
Validation:		0.92	0.93	0.93	0.92
CNN	1	0.78	1.00	0.61	0.75
	2	0.83	1.00	0.69	0.82
	3	0.81	1.00	0.67	0.80
Validation:		0.75	1.00	0.58	0.73

**Figure 6:**
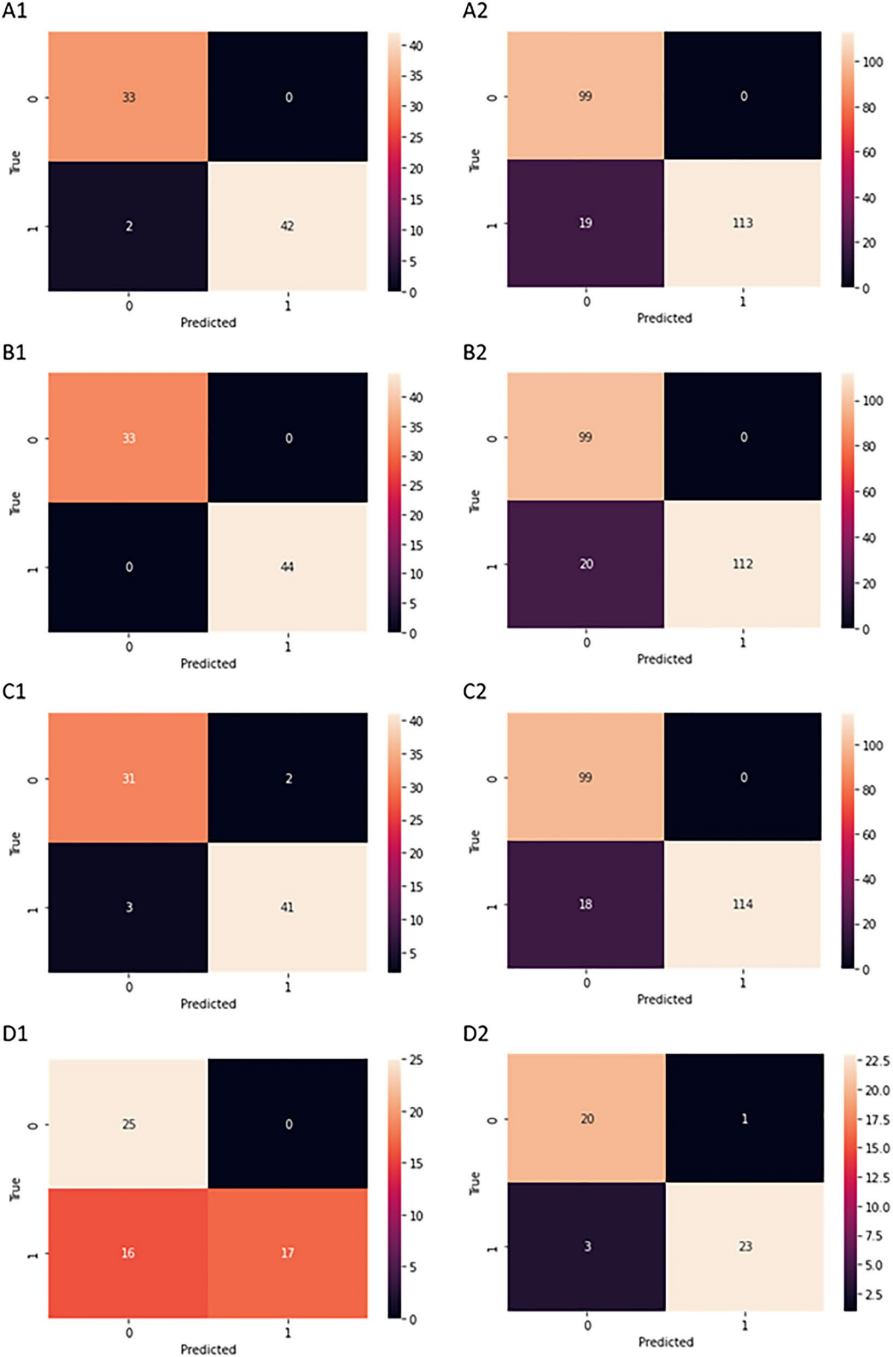
Confusion matrices for selected algorithms: A1: Random Forest test dataset fold 1, A2: Random Forest validation dataset, B1: XGBoost test dataset fold 1t, B2: XGBoost validation dataset, C1: Naive Bayes test dataset fold 1, C2: Naïve Bayes validation dataset, D1: CNN test dataset fold 1 D2: CNN validation dataset.

It can be seen that Random Forest, XGBoost, Naïve Bayes classifiers give results with accuracy above 95%, precision above 95%, recall above 95% and F1-score above 95% for training datasets. On validation, the results were as follows: accuracy above 89%, precision above 90%, recall above 90% and F1-score above 89%. Thus, the most promising results on training were obtained with XGBoost (Accuracy equals 100%, Precision equals 100%, Recall equals 100%, F1-score equals 100%). However, XGBoost did not accomplish the best results on the validation set, where the best results were obtained for Naïve Bayes (Accuracy equals 92%, Precision equals 93%, Recall equals 93%, F1-score equals 92%). The worst results were obtained for frequently used in biomedical applications Convolutional Neural Networks (CNN). In fact, here we have noticed the greatest impact of the overfitting phenomenon. It may be due to the too intensive learning process for the issue under consideration. The obtained results are presented also as confusion matrices in Fig. 6.

Additionally, to extend the model evaluation, the learning time from the training data and making predictions was measured for each algorithm. The results are presented in Table 5.

**Table 5:** Average time for training and prediction for chosen algorithms.

Algorithm	Training	Prediction
Random Forest	212 ms	15.5 ms
XGBoost	21 ms	2.08 ms
Naïve Bayes	7.54 ms	1.81 ms
CNN	5320 ms	5 ms

It can be noted that the Naive Bayes method not only gives the best results for the validation test, but also is the fastest regarding the training and prediction phases.

## Conclusions

In this study, we presented a novel approach to the analysis of data acquired by a low-coherence interferometer. The optical sensor is able to detect changes in the refractive index of samples, including the biological range of values. Hence, it can be used for measurements and initial assessment of the neoplastic cervical lesions stage. The data obtained for test liquids were acquired with a Fabry–Perot interferometer and then applied in the machine learning algorithm. Interferograms representing the optical properties of measured substances in conjunction with meta-data from the measurements are transformed into multidimensional datasets. A number of heuristics have been defined on the basis of which these datasets are constructed, taking into account their use in predictive modeling. A particularly important stage in the machine learning process was the development of an original approach to the initial processing and enrichment of data sets. Part of data was used to train the algorithm, and the other served for validation of its proper operation. The proposed solution allows for the identification and classification of healthy and sick tissues. The tested classical classifiers were characterized by high accuracy above 95%, precision above 95%, recall above 95% and F1-score above 95% for training datasets, and for validation accuracy above 89%, precision above 90%, recall above 90% and F1-score above 89%. The method we reported can be of great assistance for doctors in early cervical cancer diagnosis.

## Data Availability

The measurement data can be accessed from Open Research Data Repository: Bridge of Data under 10.34808/ax9m-cg47 and 10.34808/bt42-hj36.
